# ACL injuries in skeletally immature athletes

**DOI:** 10.3389/fped.2026.1840962

**Published:** 2026-07-29

**Authors:** Jason G. Ina, Mininder S. Kocher

**Affiliations:** 1 Division of Sports Medicine, Boston Children’s Hospital, Boston, MA, United States; 2 Department of Orthopaedic Surgery, Rainbow Babies and Children’s Hospital, Cleveland, OH, United States

**Keywords:** anterior cruciate ligament, knee, pediatric, physeal sparing, sports injury

## Abstract

Anterior cruciate ligament (ACL) injuries in skeletally immature patients are becoming increasingly prevalent. These injuries present a unique set of challenges because of the presence of open physes and the associated risk of inducing a growth disturbance with traditional ACL surgical management. This review highlights the relevant anatomy, biomechanics, epidemiology, and risk factors associated with ACL injuries in the skeletally immature population and outlines a thorough evaluation process to determine the appropriate treatment strategy for each individual patient. Although non-operative management may be appropriate in select patients, operative intervention may be best for complete ACL injuries to prevent the risk of secondary injury to associated intra-articular structures. Both physeal-sparing and physeal-respecting approaches may be considered, and the most appropriate treatment option can be determined through assessment of remaining growth. Surgical management of these injuries with the correct approach can result in low complication rates [including retear rates (6%–11%) and low growth abnormalities (1%–2%)], as well as high rates of return to sport and patient satisfaction.

## 1 Introduction

Although anterior cruciate ligament (ACL) tears were initially thought to be rare in the skeletally immature population, recent studies have demonstrated an increase in prevalence of these injuries in pediatric patients, with higher incidence rates of injury in girls compared with boys (1–4). This increased rate of ACL injuries may be attributed to the popular trend of early sport specialization in youth athletes with earlier and more frequent exposure to injury (5). Treatment of ACL injuries in this patient population can be challenging because of the nearby open physis and risk of inducing a growth abnormality with surgical treatment. On the other hand, non-operative management of complete ACL tears has been shown to have high failure rates, which can lead to secondary cartilage and meniscus injuries (6, 7). Therefore, care for these patients requires special consideration for the inherent risks and potential benefits of available treatment options.

In this narrative review, we discuss the initial evaluation, treatment options, surgical techniques, and outcomes associated with ACL injuries in the skeletally immature population. In addition, we focus on reviewing updated literature and the incorporation of new techniques such as lateral extra-articular ACL reconstruction in the skeletally immature patient population. It is crucial that the treating surgeons understand the underlying principles of treating ACL injuries in this population and how they differ from the treatment approaches used in the skeletally mature population.

## 2 ACL anatomy and biomechanics

**Figure 1:**
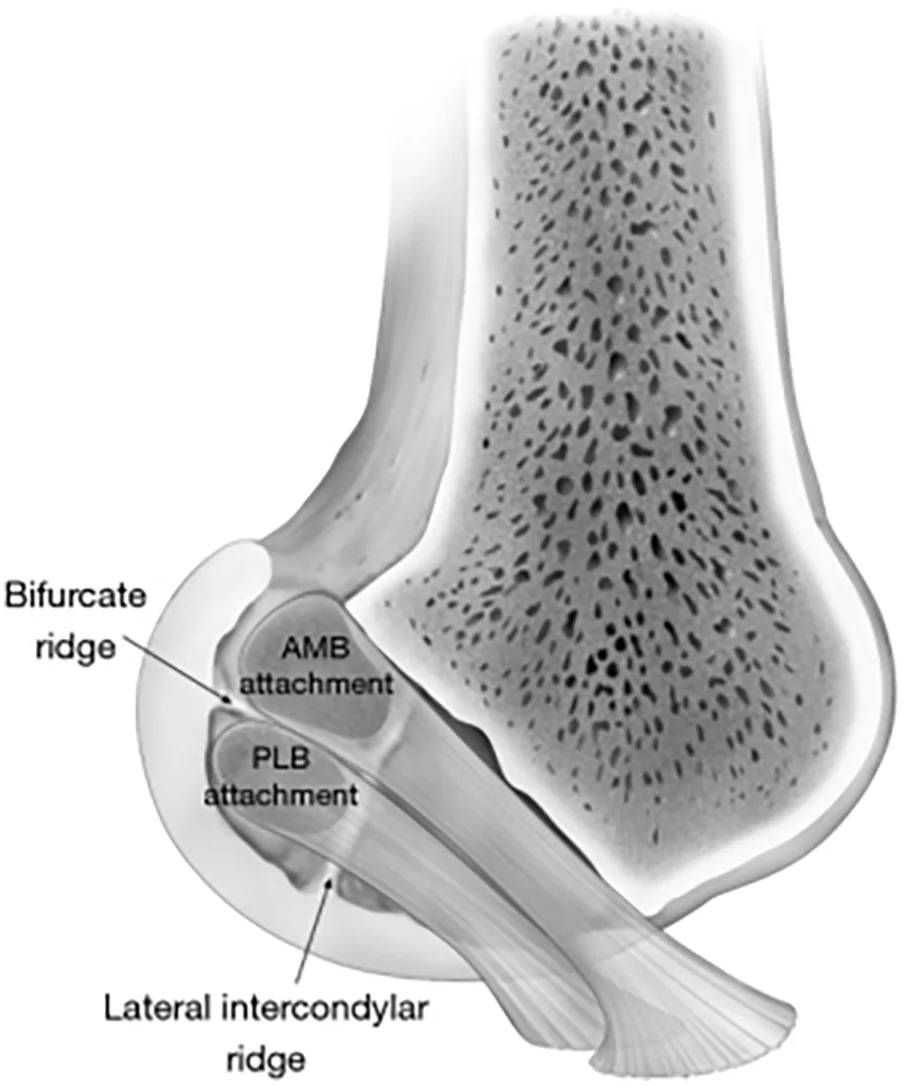
Femoral insertion of ACL bundles relative to the lateral intercondylar ridge and bifurcate ridge. Adapted from Banovetz et al. (81).

The ACL is comprised of two bundles that originate from the posteromedial aspect of the lateral femoral condyle and are named according to their relative tibial insertion (Figure 1). The anteromedial (AM) bundle originates on the femoral condyle posterior to the lateral intercondylar ridge (i.e., resident’s ridge) and proximal to the bifurcate ridge. In comparison, the posterolateral (PL) bundle originates posterior to the lateral intercondylar ridge and distal to the bifurcate ridge. Although described as distinct origins, there remains some debate regarding the overlapping origin of these two bundles (8). The AM and PL bundles traverse the intercondylar notch in an oblique fashion from posterolateral to anteromedial where they insert onto the anteromedial tibial plateau. The insertion of the ACL most commonly takes place in the form of an elliptical distribution that is closely associated with the anterior root of the lateral meniscus (9).

The intra-articular length of the ACL is variable, ranging from 22 to 41 mm, with an average length of 32 mm from the femoral origin to the tibial insertion (10–12). This wide variability of intra-articular length is likely attributable to both patient-specific anatomic variation and the relative anisometry of the ACL. With increasing knee flexion angles, the AM bundle lengthens/tightens, while the PL bundle shortens/relaxes, with a mean change in length of 4–7 mm through the knee range of motion (13, 14).

As a result of this anisometry, the function of the ACL changes through variations in the knee flexion angle. Between 0° and 45° of knee flexion, the PL bundle experiences greater *in situ* forces than the AM bundle when an anterior tibial translation force is applied, with maximum forces experienced by the PL bundle at 15° of knee flexion (15). Although the forces experienced by the PL bundle appear to be highly dependent on the knee flexion angle, the forces experienced by the AM bundle are less dependent on the knee position and remain relatively constant through knee motion (15). The anisometry of the ACL bundles can also explain the differential function of each bundle. When the AM bundle is transected, a resultant increase in anterior tibial translation is observed at higher knee flexion angles (60°–90°) (16). However, when the PL bundle is transected, increased anterior tibial translation is observed at 30° of knee flexion. The effect of PL bundle transection is further exacerbated when a combined internal tibial rotation force is applied at 0° and 30° of knee flexion, particularly when compared with both an intact knee and isolated AM bundle transection.

**Figure 2:**
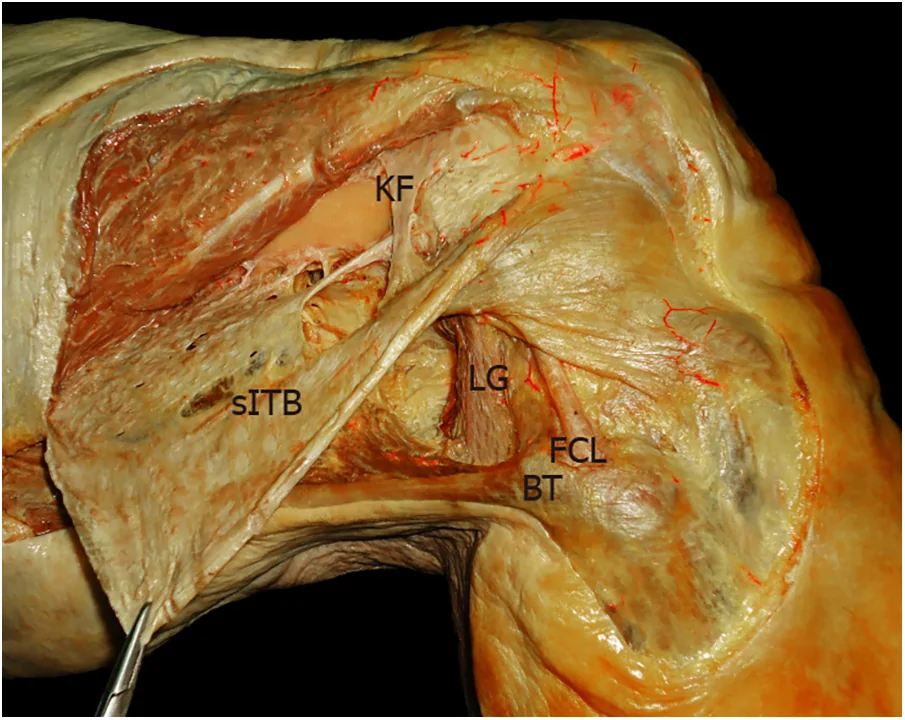
Anatomic dissection of the lateral side of a right cadaveric knee depicting a superficial iliotibial band (sITB), and Kaplan fiber (KF) insertion onto the lateral distal femur. The relationship with the structures can be seen relative to the lateral gastrocnemius muscle (LG), fibular collateral ligament (FCL), and biceps tendon. Adapted from Garcia-Mansilla et al. (82).

In addition to the ACL, the iliotibial band (ITB) makes a substantial contribution to knee stability. The portion of the ITB responsible for its contribution to knee stability consists of Kaplan fibers that attach the posterior aspect of the distal ITB to the proximal femur to the lateral femoral condyle (Figure 2) (17, 18). Although the ACL is clearly the primary restraint to anterior tibial translation, the ITB provides additional resistance to both anterior tibial translation and internal tibial rotation (19). In an ACL-deficient knee, the ITB resists approximately a third of the anterior tibial translation force and is the most substantial restraint to internal tibial rotation at knee flexion angles greater than 30° (19, 20).

## 3 Epidemiology

ACL tears in pediatric and adolescent age groups are common, with a prevalence of 121 per 100,000 person-years increasing at an annual rate of 2.3% (21). Beck et al. reported that the rates of injury were higher for high-school-aged children, peaking around 16 years for girls and 17 years for boys, with a rapid decrease in injury rate thereafter (21). When evaluating patients aged 6–14 years, they reported that the injury rate increased by 2.1% per year. However, these patients had a substantially lower prevalence of ACL injuries when compared with their high-school-aged counterparts. Similarly, a Norwegian registry study evaluated the incidence of pediatric ACL reconstruction from 2005 to 2021 and reported a 40% increase for boys and 55% increase for girls over this time period (3).

Several risk factors have been identified as being associated with an increased rate of ACL injury in pediatric patients. These risk factors can be categorized as intrinsic (non-modifiable) and extrinsic (modifiable) factors. Intrinsic risk factors include female sex, narrow intercondylar notch width, increased posterior tibial slope, generalized ligamentous laxity, and family history of ACL injury (4, 22–25). Extrinsic risk factors include neuromuscular parameters such as dynamic valgus and high abduction loads, elevated BMI, and type of sport participation (22, 26–28).

Prior studies have identified that as patient age increases, so too does the rate of concomitant meniscal, collateral, and posterior cruciate ligament (PCL) injuries in the setting of an ACL injury (29–31). A study by Pruneski et al. evaluated the prevalence and risk factors of concomitant meniscal and ligamentous injuries associated with ACL surgery in pediatric and adolescent patients over a 20-year period (1). They reported that pediatric patients (aged 5–13) had the lowest rate of concomitant soft-tissue injuries when compared with adolescent (aged 14–19) and adult (aged 20–35) patients. However, female children had increased rates of medial collateral ligament injury as well as medial meniscal injury. In addition to patient-specific factors, delayed surgical intervention—between 6 weeks and 3 months from injury—has been found to be associated with the presence of increased cartilage and meniscal injuries that require additional operative procedures (32, 33).

## 4 Clinical evaluation

Clinical evaluation of pediatric and adolescent patients presents a unique set of challenges, as obtaining a detailed history can be difficult in this population. However, it is paramount that a thorough attempt is made to elicit the mechanism of injury, timing of injury, persistence of instability, and the presence of any associated mechanical symptoms. Typically, the injury is non-contact in nature and related to a cutting or pivoting maneuver. The development of a rapid effusion at the time of injury can be suggestive of an ACL injury, as up to 65% of pediatric patients with hemarthrosis have an underlying ACL injury (34).

A physical examination is vital to the clinical assessment of a patient who presents with suspected ACL injury, as an extraordinary amount of information can be obtained through a detailed examination. Prior to evaluating the ACL, a general assessment evaluating relative lower-extremity coronal plane alignment should be performed. In addition, the knee should be examined for the presence or absence of an effusion, range of motion, and atrophy of the quadriceps muscle. When assessing the atrophy of the quadriceps, particular attention should be paid to the function of the vastus medialis oblique (VMO), as the presence of preoperative VMO arthrogenic muscle inhibition (AMI) has been identified to be a significant risk factor for the development of postoperative AMI and subsequent postoperative extension deficit (35). For ACL assessment, while Lachman's test is the most sensitive examination maneuver, the anterior drawer test should also be performed for a thorough evaluation (36). The pivot shift test also demonstrates both a high sensitivity and specificity for ACL injuries (36). However, performing this test can be difficult as it requires patient cooperation, which can be challenging in the acute setting, and may be more appropriately performed with the patient under anesthesia (37). It is also important to assess for joint line tenderness and perform a special test for meniscus pathology (i.e., McMurray's test or Apley's test) to gain an understanding of concomitant underlying intra-articular injuries. In addition, a thorough ligamentous examination should be performed to rule out the presence of a multiligamentous knee injury. A posterior drawer test can be performed to evaluate the PCL, and an assessment of coronal plane stability by applying valgus and varus stress at both 0° and 30° of flexion can help identification of associated collateral ligament and capsular injuries. If suspicion persists for either PCL or lateral collateral ligament (LCL) injury, a dial test performed in the prone position can be added to the list of physical examinations to be performed. Last, evaluation of hypermobility using the Beighton score can help identify patients who may be at a higher risk for recurrence of ACL injuries (38, 39).

## 5 Imaging

Imaging of pediatric patients who present with knee pain should begin with standard anteroposterior (AP), lateral, and patellar views. These radiographs can help in identifying traumatic injuries, including fractures, or signs of ligamentous injury, including tibial spine avulsion, lateral condyle impaction fracture, segond fracture, or arcuate sign. In addition, radiographs can be used to assess the size of the intercondylar notch, as this has been identified as a risk factor for ACL tears (40). If history and physical examination raise concerns for osteochondritis dissecans, then a notch view should be added to the initial radiographic series. When evaluating these injuries, one should also carefully assess for physeal injuries, and, if concern persists, contralateral radiographs can be obtained for comparison. If a physical examination and imaging assessment do not specify a clear reason for the presenting knee pain, then an assessment of the ipsilateral hip with AP and frog-leg views should be performed.

In addition to standard knee radiographs, standing hip-to-ankle radiographs should be obtained to evaluate lower-extremity alignment and any preexisting leg-length discrepancy. With respect to sagittal alignment, the posterior tibial slope can be evaluated by either a standing full-length lateral tibia radiograph or a lateral knee radiograph; however, full-length tibia films have been reported to be more reliable with less variation in measurement (41, 42). If pathologic malalignment is present, the addition of growth modulation can be considered at the time of surgery, as this can substantially reduce the forces transmitted to the ACL graft (43).

An assessment of remaining growth is crucial in the workup of skeletally immature patients with ACL injuries undergoing operative management to select the most appropriate ACL reconstruction technique. Although many methods exist for assessing remaining growth, including chronological age, growth charts, Tanner staging, and onset of menarche in girls, skeletal age remains the most accurate assessment method. A single posterior–anterior left-hand radiograph can be used with the Greulich and Pyle method to estimate skeletal age (44). The modified Fels knee system can also allow for accurate skeletal maturity estimation with the use of a single AP knee radiograph, which can protect the knee from additional radiation exposure associated with an additional hand X-ray (45).

**Figure 3:**
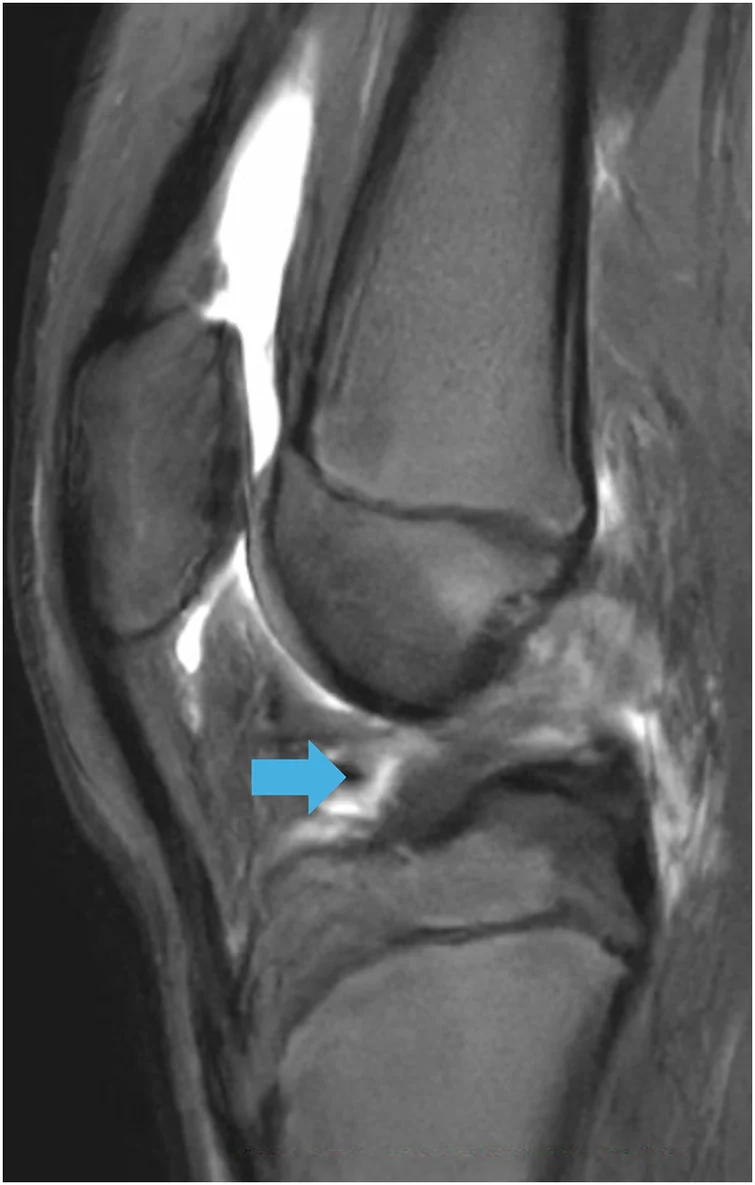
MRI findings associated with ACL tear on a T2 sagittal image of the knee. The ACL (arrow) shows abnormal takeoff with fibers that diverge from the Blumensaat line. In addition, increased signal intensity and discontinuity of the fibers can be seen at the femoral insertion.

MRI is the gold standard for evaluation of soft-tissue injuries of the knee. The 3T MRI has been shown to have high sensitivity and specificity for diagnosing ACL injuries in a skeletally immature population, despite earlier concerns about its accuracy, when compared with an adult population (46, 47). MRI findings consistent with ACL injury include bone bruising of the lateral femoral condyle and lateral tibial plateau consistent with a pivot-shift injury, discontinuity of the fibers of the ACL, and abnormal orientation of the fibers that diverge from the Blumensaat line (Figure 3) (48, 49). A thorough evaluation of the MRI should also be performed to assess for signs of associated pathology, including cartilage injuries, meniscus tears, and multiligamentous injuries. Special consideration should be given to meniscus tears associated with ACL injuries, such as ramp lesions (associated with bone marrow edema in the posterior aspect of the medial tibial plateau near the semimembranosus insertion) and lateral meniscus posterior root tears (50, 51). Of note, MRI in the pediatric population has a high false-positive rate for meniscal tears, which is likely secondary to the increased vascularity of the pediatric meniscus, which can often be incorrectly interpreted as intrasubstance degeneration or tearing.

## 6 Treatment

### 6.1 Non-operative management

Historically, ACL injuries in skeletally immature patients were managed non-operatively with activity modification and physical therapy in an attempt to delay operative management until patients came close to attaining skeletal maturity. Unfortunately, non-operative management of complete ACL injuries in this population was found to result in an unacceptably high rate of subsequent injuries due to recurrent instability, including cartilage damage and secondary meniscus tears (6, 7, 52).

In the appropriate setting, non-operative management can be a reasonable treatment option for skeletally immature patients with ACL tears. Kocher et al. performed a retrospective review of 45 skeletally immature patients who were 17 years of age or less with a partial tear of the ACL identified on MRI and confirmed with arthroscopy. These patients were treated with non-operative intervention (53). At a minimum 2-year follow-up, 14 patients (31%) underwent subsequent ACL reconstruction. Findings associated with subsequent ACL reconstruction included grade B pivot shift, older skeletal age, tears greater than 50% of the ACL, and posterolateral bundle tears. Notably, patients who remained in the non-operative treatment arm but had tears greater than 50% of the ACL and posterolateral bundle tears had lower patient-reported outcome measures. In addition, Hannon et al. performed a retrospective review of patients aged 8–18 years diagnosed with a partial ACL tear and reported that 25% of patients ultimately underwent conversion to ACL reconstruction at a median survival time of 11.4 months after returning to sports (54).

Successful non-operative management in appropriately selected patients is dependent on a robust rehabilitation protocol. Typically, patients are placed in a hinged knee brace for 12 weeks with touchdown weight-bearing maintained for 6–8 weeks. With regard to range of motion, passive terminal extension is restricted for the first 6 weeks after injury, and active terminal extension is restricted to 12 weeks postinjury. Initial exercises focus on quadriceps activation, patellar mobilization, range of motion, hamstring, abductor, and core muscle strengthening, and closed-chain exercises for the first 12 weeks, with open-chain activities started at 12 weeks. Return to sports and active play can be permitted at 4–6 months depending on outcomes of strength and functional testing. Although the utility of a functional ACL brace is poorly understood, we recommend the use of a functional knee brace for at least 1 year for cutting and pivoting activities (55).

### 6.2 Surgical management

Surgical management is recommended for skeletally immature patients with complete ACL tears due to the sequelae of persistent knee instability and the resultant low rate of return to sport (7). The benefits of improved stability and decreased secondary injuries achieved with earlier surgical management should be weighed against the risk of growth disturbance when performing ACL reconstruction in patients with substantial remaining growth. Therefore, it is important to consider skeletal age when determining which surgical technique is best suited for each individual patient. Regardless of the ACL reconstruction method chosen, certain physeal-respecting principles should be followed (Table 1). Adherence to these principles limits iatrogenic injury to the cross-sectional area of physis, which has been associated with a resultant growth disturbance in the distal femoral physis (56).

**Table 1:** Physeal-respecting principles for ACL reconstruction.

Physeal-respecting principle
No hard fixation (i.e., interference screws) should cross the physis because of an increased risk of growth disturbance. No bone (i.e., bone block from bone-patellar tendon-bone graft) should cross the physis because of an increased risk of growth disturbance. Drill holes across the physis should be as small and central in the physis as possible. Oblique drill holes violate a larger cross-sectional area of the physis compared with perpendicular drill holes of a similar diameter. A tensioned soft-tissue graft in a bone tunnel can induce a growth abnormality. Excessive dissection about the posterolateral aspect of the femoral physis or aggressive notchplasty can violate the perichondral ring of LaCroix and subsequently induce growth disturbance.

**Figure 4:**
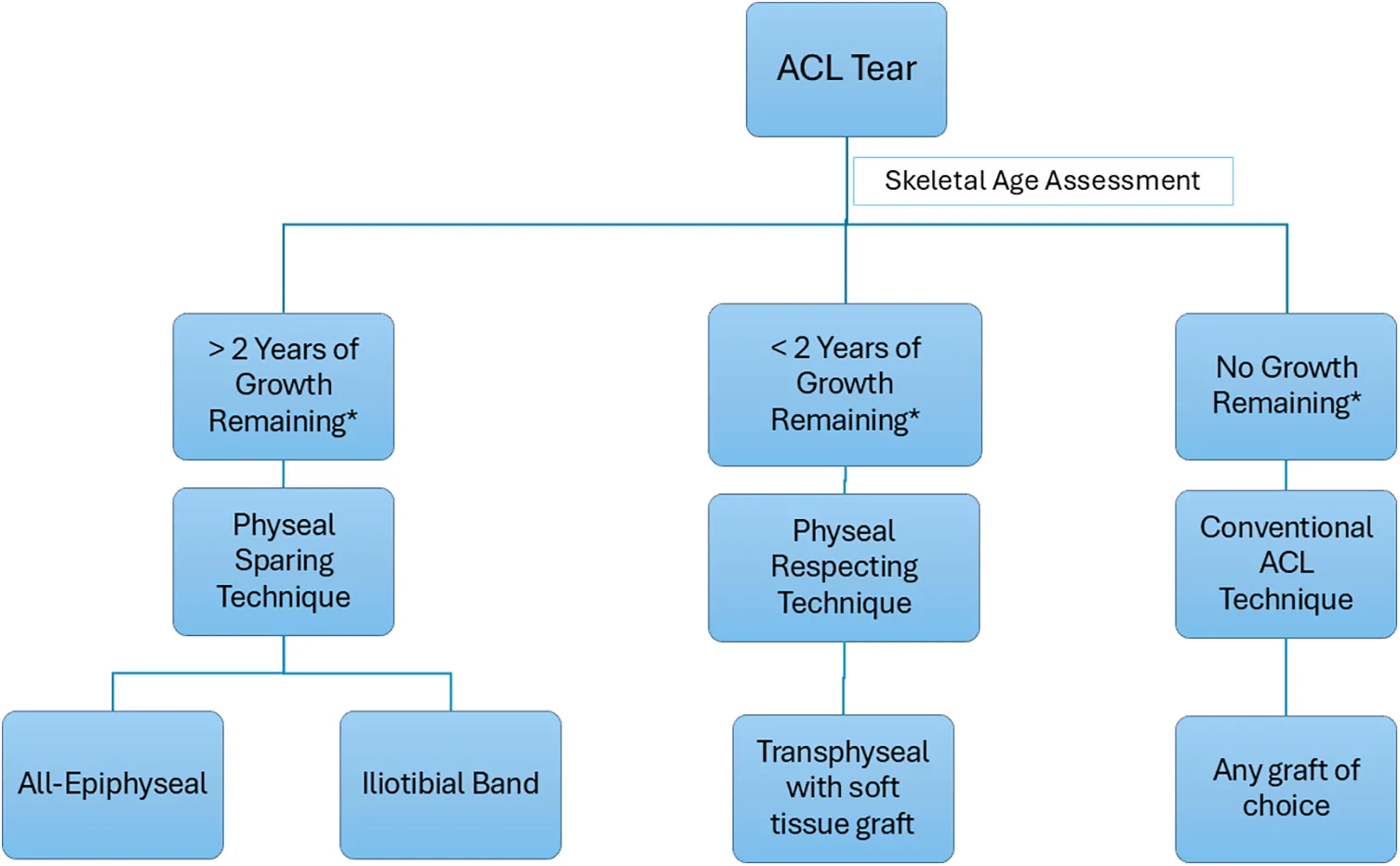
Treatment algorithm for ACL tear reconstruction techniques in a skeletally immature athlete. *Remaining growth is determined based on skeletal age assessment separately for males and females, with the assumption that males complete growth at the skeletal age of 16 and females complete growth at the skeletal age of 14.

Options for ACL reconstruction of skeletally immature individuals include physeal-sparing techniques such as the iliotibial (IT) band combined intra-articular and extra-articular reconstruction and all-epiphyseal ACL reconstruction. In addition, physeal-respecting techniques include a partial transphyseal (all-epiphyseal or over-the-top femoral technique with the transphyseal tibial technique) or transphyseal ACL reconstruction with a soft tissue graft (quadriceps tendon or hamstring tendon). The decision to proceed with which reconstruction option is generally dictated by the number of years of remaining growth left as determined by skeletal age (Figure 4). However, additional considerations such as overall knee size can also influence the decision-making process as patients with more than 2 years of growth remaining but with a relatively small femoral epiphysis may make the case for an all-epiphyseal ACL reconstruction challenging. In general, patients with more than 2 years of remaining growth as determined by bone age assessment undergo a physeal-sparing technique, and patients with less than 2 years of growth remaining undergo a physeal-respecting technique. A cutoff of 2 years of remaining growth is chosen based on a consensus that this would limit the overall effect of growth disturbance, should it occur, and reduce the need for a secondary procedure to address the growth abnormality, should it occur with a physeal-respecting technique. In adolescent patients approaching skeletal maturity with closing physes, a conventional transphyseal ACL reconstruction technique can be considered. In patients with approximately 2 years of skeletal growth remaining, surgical decision-making is based on a multifactorial assessment that includes activity level, sex, and, for girls, the onset of menarche. In general, we prefer to proceed with a physeal-sparing IT band technique given its high clinical success rate and lower risk of growth disturbance when compared with all-epiphyseal or transphyseal techniques in this particular population.

The addition of a lateral extra-articular procedure (LEAP) has been demonstrated to decrease graft rupture rates after ACL reconstruction (57, 58). In particular, these findings are consistent across pediatric and adolescent literature, with significant reduction in graft rupture rates in skeletally immature patients undergoing ACL reconstruction with the addition of a LEAP. A consensus statement has recommended the addition of a LEAP for active patients aged 25 years and younger (59). Additional recommendations for a LEAP include patients undergoing ACL reconstruction with hamstring autograft, knee hyperextension, skeletally immature status, and grade 3 pivot shift and/or Lachman test. By design, IT band ACL reconstruction incorporates a LEAP, and therefore, no additional procedure needs to be performed in these patients. However, other physeal-sparing (all-epiphyseal) or physeal-respecting (transphyseal with a soft tissue graft) techniques may benefit from the addition of a LEAP in appropriately selected patients. Although the aforementioned consensus statement would recommend a LEAP for skeletally immature patients, we would emphasize judicious utilization of this adjunct procedure as the long-term consequences are not well understood. However, we would continue to recommend its use in all skeletally immature patients undergoing a hamstring autograft ACL reconstruction.

When adding a LEAP to ACL reconstruction in a patient with significant growth remaining, the surgeon needs to be cautious about the placement of femoral-sided fixation to avoid both the physis and the ACL femoral tunnel. The proximity of the lateral extra-articular tenodesis (LET) insertion site to the distal femoral physis does raise a concern for iatrogenic growth injury as a potential complication. Despite this risk, prior studies have reported low rates of growth abnormalities after the addition of LET, ranging from 0.63% to 2.6% (60–62). In addition, while concern remains for lateral compartment overconstraint after the addition of a LEAP, supporting data have been limited to biomechanical studies in which the LEAP was secured with the leg in relative external rotation, and this finding has not been demonstrated in clinical studies (63, 64). Therefore, long-term studies are needed to better understand the benefits and potential consequences of the addition of a LEAP to ACL reconstruction in the skeletally immature population.

## 7 Surgical techniques

The setup and patient positioning is relatively similar across the numerous ACL reconstruction techniques. The patient is positioned supine on a standard operating table with a tourniquet on the operative extremity. A lateral post is positioned roughly four finger-breadths proximal to the superior pole of the patella. Prior to proceeding with any ACL reconstruction procedure, an examination should be performed with the patient under anesthesia to evaluate the stability of the knee and determine whether any additional procedure should be considered. If questions are raised about the integrity of the ACL after the examination, it is reasonable to consider an arthroscopic evaluation prior to proceeding with graft harvest to confirm the diagnosis.

**Figure 5:**
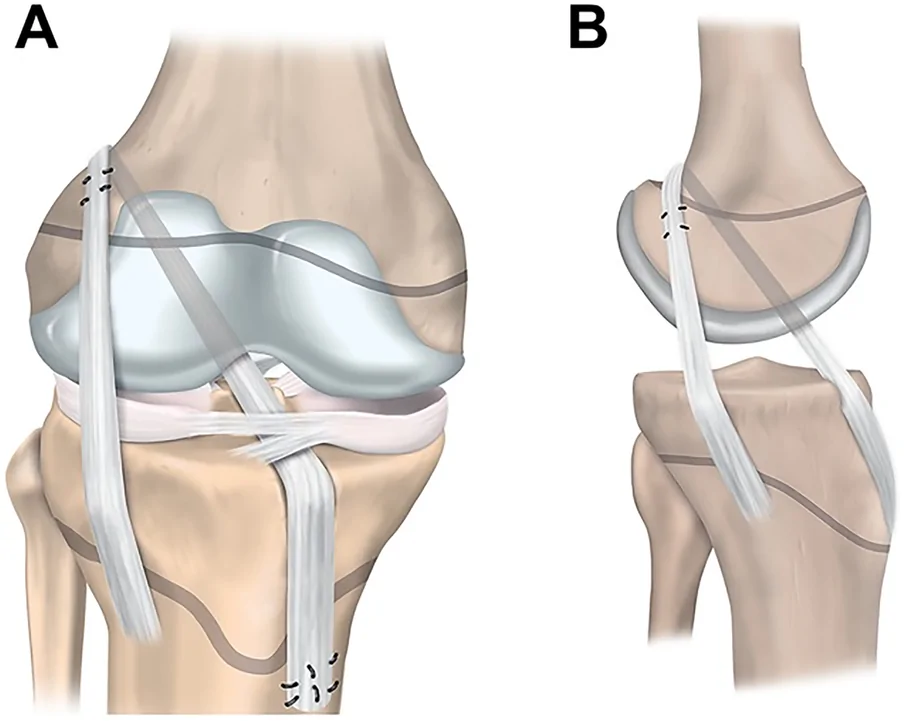
An illustration depicting a physeal-sparing combined intra-articular and extra-articular anterior cruciate ligament reconstruction with an over-the-top technique utilizing the iliotibial band with **(A)** anterior and **(B)** lateral views. Adapted from Ardern et al. (83).

**Figure 6:**
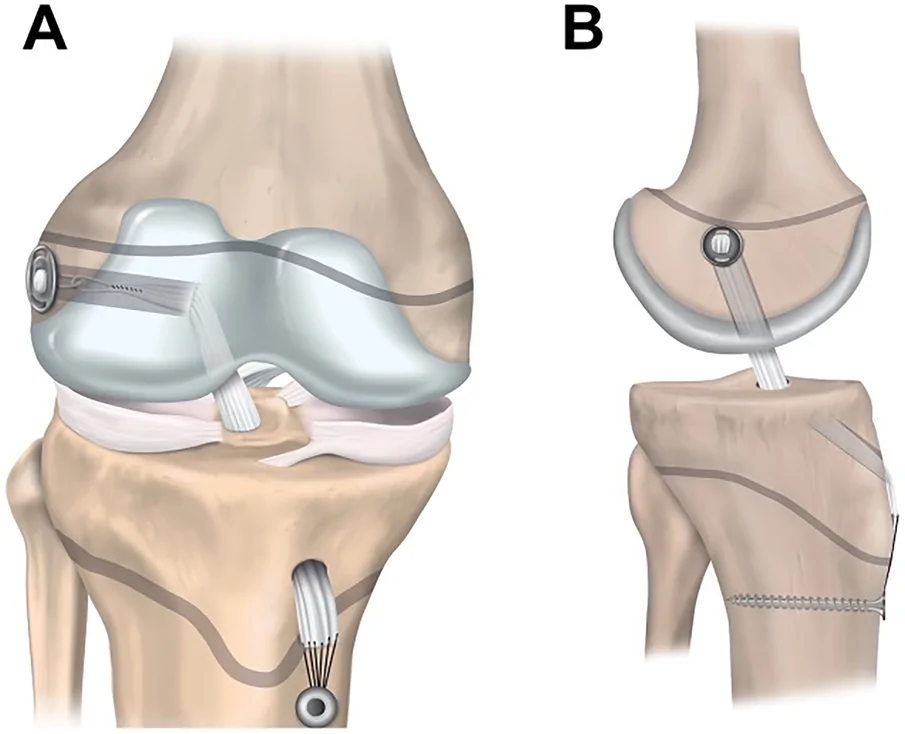
An illustration depicting an all-epiphyseal anterior cruciate ligament reconstruction utilizing a soft-tissue graft with **(A)** anterior and **(B)** lateral views. Adapted from Ardern et al. (83).

In the following, we describe two physeal-sparing ACL reconstruction techniques, namely, the combined intra-articular and extra-articular IT band reconstruction (Figure 5) and all-epiphyseal reconstruction (Figure 6). Although the physeal-respecting technique can also be utilized, it is similar to standard ACL reconstruction in adults, with the exception of utilization of all soft tissue grafts and adherence to physeal-respecting principles (Table 1). Numerous techniques can be utilized for the physeal-respecting approach so long as the relevant principles are taken into consideration.

### 7.1 Physeal-sparing combined intra-articular and extra-articular reconstruction with iliotibial band

Although the IT band technique has the advantage of avoiding the tunnel in a skeletally immature knee, it is a non-anatomic technique (65). When positioning and draping for IT band ACL reconstruction, one needs to ensure that the tourniquet is placed as proximally as possible and that the proximal thigh is prepped into the field to allow access to the proximal IT band as needed. The procedure begins with the harvest of the IT band graft. An oblique 6-cm incision is made from the level of the joint line to the anterior aspect of the IT band, centered over the lateral epicondyle. The anterior and posterior borders of the IT band are marked, and a 2-cm-wide graft is harvested. Notably, the graft should be eccentric in the IT band, with the center over the more posterior aspect of the IT band. A knife is used to create a nick in the IT band graft over the posterior and anterior aspects proximal to the lateral epicondyle to avoid injury to the deeper LCL. A meniscotome is then utilized to incise the IT band proximally in line with the fibers. The goal is to harvest a graft that is at least 15 cm from the level of the lateral epicondyle. However, the length of the desired graft may vary based on the size of the patient. The IT band is then detached proximally either with the use of a curved meniscotome, a tendon stripper, or through a proximal counter-incision. The IT band is then incised distally, with care taken to avoid injury to the deeper LCL and lateral capsule, and the incision is extended to the level of the femoral articular cartilage, which can be determined by palpation. In addition, this distal incision of the IT band will release any tethering between the IT band and the patella and prevent lateral patellar maltracking through tensioning of the graft. The free proximal end of the graft is then tubularized with a No. 5 non-absorbable suture.

**Figure 7:**
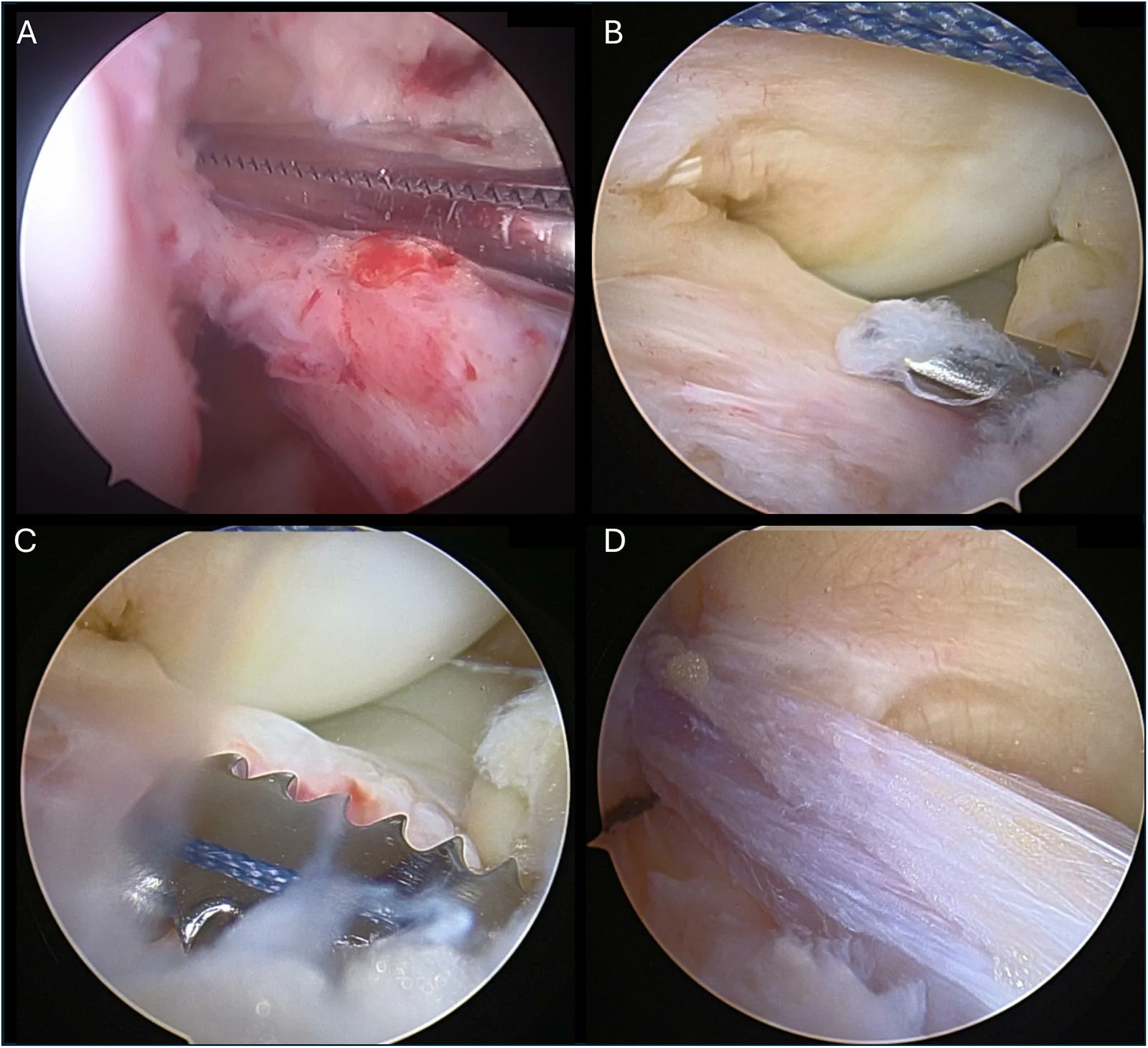
Arthroscopic photographs highlighting the key intra-articular steps when performing a physeal-sparing combined intra-articular and extra-articular reconstruction with an iliotibial (IT) band autograft. **(A)** A full-length clamp is placed from the anteromedial portal through the remnant ACL in the over-the-top position behind the lateral femoral condyle. The remnant ACL fibers are left intact to function as a sling for the IT band graft to prevent graft slippage behind the lateral femoral condyle. **(B)** The graft sutures are then passed out of the anteromedial portal for later graft passage. A curved clamp is then passed proximally from the tibial fixation site on the anteromedial tibia into the joint under the intermeniscal ligament to establish the extra-articular path of the IT band graft. **(C)** A rasp is passed in the same path established by the curved clamp to debride a path for the IT band graft. This not only helps posterize the ACL graft but also promotes intra-articular healing of the graft. **(D)** The IT band graft is then passed and fixed in its extra-articular path positioned at the lateral femoral condyle and anteromedial tibia.

A diagnostic arthroscopy is then performed in standard fashion using a standard anterolateral arthroscopy portal. The anteromedial arthroscopy portal is positioned slightly more medial and just proximal to the anterior horn of the medial meniscus to obtain the appropriate trajectory for later graft passage. Meniscal and cartilage injuries are then addressed as indicated. The native ACL is left intact as a supportive sling that will be used to prevent the IT band graft from slipping around the posterior aspect of the lateral femoral condyle into the lateral tibiofemoral joint. A Kelly clamp with a preloaded passing suture is then passed from the anteromedial portal, above the native ACL origin, behind the lateral femoral condyle, and out of the lateral capsule, taking care to avoid injury to the perichondral ring of the LaCroix (Figure 7A). Ideally, the clamp should exit the capsule just posterior to the lateral femoral condyle within the IT band harvest site. The passing suture is then used to shuttle the free end of the IT band graft into the joint and out of the anteromedial portal.

Attention is then directed to the tibial fixation site. A 2–3-cm incision is made 1–2 cm medial to the tibial tubercle, and dissection is carried down to the level of the periosteum. Care should be taken not to dissect proximally to the superior aspect of the tibial tubercle to avoid injury to the proximal tibial physis. The periosteum over the anteromedial tibia is then sharply incised and elevated. A trough is created for later graft fixation. A curved clamp is passed from the proximal extent of this position into the joint under the intermeniscal ligament (Figure 7B). Next, a rasp is replaced with this clamp to create a trough in the anteromedial tibia for the IT band graft (Figure 7C). The free end of the IT band graft is then passed through the joint, under the intermeniscal ligament, and out of the anteromedial tibial incision (Figure 7D).

Attention again shifts to the extra-articular portion of the graft on the femoral side. With the knee in neutral rotation, 90° of flexion, and tension on the graft, the graft is sutured to the periosteum of the distal femur. Then, the graft is secured with suture fixation to the periosteum of the tibia. The knee is then checked for stability by performing a Lachman test and is taken through a full range of motion to ensure that both full flexion and extension are achieved.

### 7.2 Physeal sparing with all-epiphyseal fixation

The all-epiphyseal technique restores the anatomic ACL origin and insertion, while keeping the fixation entirely within the epiphysis (66). However, intraoperative fluoroscopy is required to ensure that tunnel placement remains distal to the femoral physis. For this technique, either a standard quadriceps tendon or a hamstring tendon autograft can be utilized and prepared with adjustable-loop suspensory fixation devices (however, fixed-loop suspensory fixation can also be utilized with this technique). Care should be taken to ensure that the graft length is not larger than the intra-articular distance plus the femoral and tibial socket depth.

After a standard diagnostic arthroscopy is performed and all chondral and meniscal injuries are addressed, the remnant ACL is debrided using a shaver or arthroscopic biters. A notchplasty can be performed, but it should be minimal in size to avoid iatrogenic injury to the perichondral ring. The femoral socket is then positioned using an outside-in drill guide placed through the lateral portal at the location of the native ACL footprint. Dissection is then carried out to the lateral epicondyle, and a guide pin is passed through the femoral guide (typically set to 85°) to its intra-articular position. The pin is then left in place and attention is turned to the tibial tunnel. The tibial guide is then inserted into the anteromedial portal on to the tibial ACL footprint and an incision is made over the proximal tibial just proximal and medial to the tibial tubercle. The tibial tunnel guide pin is placed to its intra-articular position and left in place. Fluoroscopy is used to confirm the all-epiphyseal location of the guide pins.

Next, a retro-reamer is used over each guide pin to create femoral and tibial sockets. When reaming, it is important to take note of the depth of each socket so that the graft can be appropriately sized to prevent graft–socket mismatch. The sockets are then examined arthroscopically to ensure that the physis has not been violated. Passing sutures are placed through the sockets and out of the anteromedial portal for later graft passage. Prior to passing the sutures out of the medial portal, the medial portal should either be enlarged or a cannula should be placed to assist with graft passage to prevent the formation of a soft-tissue bridge and facilitate suture management.

The femoral side of the graft is first passed through the medial portal and into the femoral socket, with the suspensory fixation device secured on the lateral femoral epiphysis. The tibial side of the graft is then passed intra-articularly into the tibial socket, and the suspensory fixation device is secured on the anteromedial tibial epiphysis. Final tensioning is performed on the femoral-sided suspensory fixation. The knee is then placed in full extension, a posterior drawer is applied, and the tibial-sided adjustable loop suspensory fixation device is finally tensioned.

### 7.3 Postoperative care

Postoperative rehabilitation protocols for IT band ACL reconstruction and all-epiphyseal reconstruction have notable differences. We recommend protected (touch-down) weight-bearing for the first 6 weeks after IT band ACL reconstruction, accompanied by a slow progression of the range of motion (0°–30° for weeks 0–2, 0°–60° for weeks 2–4, and 0°–90° for weeks 4–6) with the use of a hinged knee brace. For the all-epiphyseal technique, we recommend 4 weeks of protected (touch-down) weight-bearing, with the range of motion permitted from 0° to 90° in the first 2 weeks and then progressing to a full range of motion thereafter. The slower progression in the case of the IT band technique is attributed to the lack of tunnels and all-suture fixation of the graft.

After the initial rehabilitation period, a progressive supervised rehabilitation is recommended, consisting of range-of-motion exercises, patellar mobilization, proprioception exercises, and closed-chain strengthening for the first 3 months. Additional modalities such as blood flow restriction therapy, pool therapy, and electrical stimulation can be utilized based on access and therapist preference. Patients can then progress to a running program with the initiation of straight-line jogging, plyometric exercises, and subsequently sport-specific exercises as tolerated. Return to sport is permitted at a minimum of 9 months once the patient has achieved full range of motion, has at least 90% symmetry in lower-extremity strength, and can perform a series of functional tests—including hop and balance tests—within 90% of the uninjured extremity.

## 8 Outcomes

Physeal-sparing ACL reconstruction has been shown to provide excellent functional outcomes in the skeletally immature population. Kocher et al. performed a retrospective study of 237 patients who underwent ACL reconstruction utilizing an IT band autograft, with a mean follow-up of 6.2 years (67). They reported a graft rupture rate of 6.6% at an average of 33.5 months. Those who did not sustain a graft rupture reported excellent patient-reported outcome measures, with a mean Pedi-IKDC score of 93.3 and a Lysholm score of 93.4. Notably, no limb-length discrepancy or angular deformity was observed. Similar results have been reported from other institutions, with a study by Willimon et al. reviewing 22 knees undergoing IT band ACL reconstruction, with a minimum 1-year follow-up. Patients reported excellent satisfaction scores, with a Pedi-IKDC score of 96.5 and a Lysholm score of 95 (68). Although they did report a slightly higher revision rate of 14%, there were no occurrences of angular deformity or limb-length discrepancy. The all-epiphyseal ACL reconstruction technique has also demonstrated acceptable clinical results, with a 10.7% rate of graft rupture and a 2% rate of arthrofibrosis requiring manipulation under anesthesia (69). However, when comparing the different physeal-sparing ACL reconstruction techniques, IT band ACL reconstruction has been found to have a lower rate of growth disturbances, angular deformity, and graft rupture when compared with the all-epiphyseal technique (70).

For patients nearing skeletal maturity, a physeal-respecting techniques—with either partial transphyseal or complete transphyseal reconstruction with soft tissue grafts—remain reasonable treatment options. Partial transphyseal and complete transphyseal reconstruction techniques have similar graft tear rates and all-cause reoperation rates (71). Although the potential for growth arrest is still present with the partial transphyseal technique, the severity of the disturbance observed is often well tolerated with a limited need for secondary intervention (72–74).

A few studies have directly compared the outcomes between physeal-sparing and physeal-respecting ACL reconstruction techniques, as the indications for these two techniques differ based on patient skeletal age. Pagliazzi et al. performed a meta-analysis of 49 studies, comparing outcomes between complete transphyseal, partial transphyseal, and physeal-sparing ACL reconstruction in patients under 20 years of age (75). Although they reported no difference in malalignment or IKDC scores, there was a difference in laxity, with the complete transphyseal technique demonstrating maximum laxity and the physeal-sparing technique demonstrating the least laxity. Similarly, Tahir et al. performed a meta-analysis of 37 studies, comparing functional and radiologic outcomes among transphyseal, partial transphyseal, and physeal-sparing ACL techniques in patients aged 10.5–16.4 years (76). They reported the highest graft rupture rate at 9.8% in the partial transphyseal group, followed by 8.1% in the physeal-sparing group and 6% in the transphyseal group.

With regard to growth disturbance, Fury et al. performed a systematic review to determine the incidence and reporting of growth disturbance after ACL reconstruction in skeletally immature patients (77). They reported a 2.1% rate of leg-length discrepancy greater than 10 mm postoperatively, with only 0.5% of patients having a leg-length discrepancy larger than 2 cm. The rate of angular deformity was less, with only 1.3% of patients reporting a postoperative angular deformity of more than 5°. The results of this study highlight the overall low incidence of growth-related complications associated with ACL reconstruction in skeletally immature patients. When comparing the rates of growth-related complications based on the ACL reconstruction technique, Tahir et al. reported the highest rate of growth disturbance with the partial transphyseal technique and the lowest with the complete transphyseal technique (76). However, this study may have some selection bias, as patients who underwent complete transphyseal ACL reconstructions were typically close to attaining skeletal maturity or had already attained skeletal maturity. In addition, the physeal-sparing technique was a mix of all-epiphyseal and IT band techniques, which have different rates of growth arrest.

A few studies have evaluated return to sport after ACL reconstruction in skeletally immature individuals. Dekker et al. performed a retrospective review of 112 patients aged 18 years or less who underwent ACL reconstruction, with a minimum 2-year follow-up (78). Although they reported a 91% rate of return to sport after ACL reconstruction, the number of sports in which the patients participated postoperatively decreased compared with the figures in preoperative sport participation. In addition, the only significant predictor for a second ACL injury was time to return to sport, with a slower return demonstrating a protective effect.

There has been increasing awareness on the influence of psychological readiness on a patient's ability to return to sport after ACLR. Psychological readiness—as determined by the ACL-Return to Sport after Injury (RSI) score—has been associated with return-to-sport testing metrics (79). In addition, other metrics such as age, sex, and patient-reported outcomes (such as IKDC scores) have been found to correlate with ACL-RSI scores (79, 80). These findings highlight the association between psychological recovery and physical recovery after ACL surgery.

## 9 Conclusion

ACL injuries in skeletally immature patients are occurring with increasing frequency. Although the number of associated injuries is lower than those in skeletally mature patients, one needs to be diligent to evaluate for underlying concomitant pathology. A physical examination is vital in the assessment of these injuries, both in the clinic and under anesthesia. MRI techniques show improved ACL tear detection rates in the younger population. Although a role exists for non-operative management in partial-thickness ACL injuries, complete ACL injuries have a high rate of failure with conservative treatment, and therefore, patients with such injuries should undergo surgical intervention to reduce the risk of secondary cartilage and meniscal injuries caused by persistent knee instability. ACL reconstruction techniques in skeletally immature patients should be dictated by remaining growth, with both physeal-sparing and physeal-respecting approaches as reasonable options. Both physeal-sparing and physeal-respecting techniques have low complication rates, high return-to-sport rates, and high patient-reported outcome measures. Although the rate of growth disturbance is low, regular screening should still be performed for angular deformity and leg-length discrepancy, with treatment as indicated.
