# Correction: ACL injuries in skeletally immature athletes

**DOI:** 10.3389/fped.2026.1955133

**Published:** 2026-08-28

**Authors:** Jason G. Ina, Mininder S. Kocher

**Affiliations:** 1 Division of Sports Medicine, Boston Children’s Hospital, Boston, MA, United States; 2 Department of Orthopaedic Surgery, Rainbow Babies and Children’s Hospital, Cleveland, OH, United States

**Keywords:** anterior cruciate ligament, knee, pediatric, physeal sparing, sports injury

Error in figure/table

The figures were in the wrong order in the online version of this paper. The images for figure 5 and 6 have been swapped. Figure 5 has the correct caption and placement in the text, but this image should be switched with the Figure 6 image. Similarly, Figure 6 has the correct caption and placement in the text, but the image should be switched with the figure 5 image. The order has now been corrected.

The original version of this article has been updated.

